# Nursing the colonised: the politics of representation of the Western nurse in plague-stricken Bombay

**DOI:** 10.1017/mdh.2026.10061

**Published:** 2026-07

**Authors:** Rinu Koshy

**Affiliations:** Department of Humanities and Social Sciences, Indian Institute of Technology Bombay, Mumbai, India

**Keywords:** Colonial nursing, Bombay plague, Non-military Western nurse, Victorian periodicals, Women’s letter-writing, Colonial medicine

## Abstract

The 1896 Bombay plague outbreak prompted the colonial government to recruit trained British nurses from England to serve the afflicted Indians of the Presidency. Studying this relatively under-explored aspect of British colonial nursing, this paper examines the politics of representation of the Western, non-military nurses serving the colonised Other through nineteenth-century periodical accounts and personal letters of a nurse stationed in Bombay. Owing to the popularity of British periodicals and the significant role they played in shaping public debates in the Metropole, periodical plague literature portrayed Western nurses as spokespersons for the Empire’s benevolent rule in Bombay. Contrarily, the intimacy and confidentiality of letter-writing allowed nurses to offer a more nuanced and critical account of life and work in Bombay. The paper contends that the non-military Western nurse’s medical career, mobility, and financial stability framed her multidimensional identity, which was further defined by the intersecting issues of race, class, gender and culture she encountered in Bombay. Comparing their varied portrayals in the periodicals and the letters, the paper argues that the politics of representation of women’s lives were influenced by both their sociopolitical subjectivities and the narrative forms through which they articulated their experiences.

## Introduction

Scholars of the British Empire have documented the diverse roles played by British women in actively shaping imperial ideologies both in the colonies and in the Metropole during the nineteenth century. Examining a selection of British women’s writings from across colonial contexts, including accounts by wives of British officers, women missionaries, female reformers, and independent women travellers, Nupur Chaudhuri and Margaret Strobel observe that these women displayed differing degrees of complicity with and resistance to the imperial ideology and enterprise.^1^ They argue that the varied standpoints maintained by these women on British imperialism can be best understood through the entangled and intersecting structures of race, culture, gender, and class that these women both navigated and perpetuated.^2^ Ironically, the same imperial ideologies of race, gender, nation, and culture also afforded them opportunities to challenge certain patriarchal restraints of the British society.^3^ It is within this discursive framework of British women as imperial agents, shaped by their multi-dimensional identities, that this paper examines the Western nurse who served the colonised Indians in nineteenth-century plague-stricken Bombay.

Colonial nursing in its various forms became prevalent in the British Empire in the nineteenth and twentieth centuries. In the aftermath of the Crimean War and the Boer War, trained nurses were recruited from England and sent to the British colonies to nurse wounded and convalescent soldiers.^4^ Contrary to war nursing, nursing in settler colonies like New Zealand and Australia was a rather permanent venture, for the intention was to build an organised medical care system to cater to the future British settlements there.^5^ Besides these, European nurses were also recruited to serve the colonised populace in the wake of emergencies like epidemic outbreaks.^6^ While imperial war nursing and settler nursing have been well-researched, the roles of British nurses serving the colonised in the peripheries remain relatively under-explored. Studying the lives and experiences of non-military nurses in the colonies is valuable, for they showcase the multi-directional exchanges in the contact zones made possible by colonial medical ventures.^7^

This paper investigates the politics of representation of the Western nurses serving the populace in nineteenth-century colonial Bombay amidst the controversies surrounding the British government’s management of the bubonic plague outbreak of 1896. Attempting to trace the non-military nurse, who is invisible in histories of nursing in colonial India, this paper relies on the Victorian periodical literature on the Bombay plague and the personal letters of a nurse, Miss Hester May Dowson, who worked in the government plague hospital in Bombay. Comparing the presentations of the non-military nurse in these two textual forms, it demonstrates the varied portrayals of their engagements with the populace and their conflicts with the imperial ideology. The paper argues that while the periodicals employed the Western nurse as a trope to disseminate imperial propaganda, the letters record her ambivalent and vulnerable position in the colonies, straddling issues of race, gender, class, and cultural alienation in the subcontinent.

## The Bombay plague epidemic: Colonial governance and Indian responses

When the plague hit colonial Bombay in 1896, the city had already gained a global reputation as the busiest port of British India.^8^ Therefore, the rapid transmission of the disease and the alarming mortality rates from Bombay attracted wide global censure. For fear of facing trade bans, the government was eager to frame plague as an endemic disease that originated from the city’s filthy quarters of the poorer Indians.^9^ Consequently, the government organised massive cleaning campaigns to whitewash the streets, buildings, and drains with carbolic acid, often evacuating its inhabitants without their consent. Additionally, the enactment of the Epidemics Act in February 1897 gave the governmental plague officials unrestricted power to detain and segregate plague suspects, and to destroy their houses and properties suspected of harbouring the disease-causing germs. Public physical searches were conducted on the passengers travelling by rail, road, and sea, and those suspected of being infected were forcibly hospitalised.^10^ Finally, reinforcing Western medicine and anti-plague vaccination as the sole means of surviving the plague, the government denigrated and denounced the indigenous practitioners of traditional medicine and faith healing.^11^

The execution of the government’s interventionist anti-plague measures, argues David Arnold, not only exposed the bodies of the residents of Bombay to the ‘gaze’ of Western medicine but also to its physical touch – an intrusion of the greatest concern to a society wherein touch connoted possession or pollution.^12^ Therefore, the intrusive plague policies that ‘assaulted’ their bodies sparked public resentment against the colonial government.^13^ Fearing the governmental measures, the poorer inhabitants fled the city in large numbers. Those who stayed back either hid the sick or the dead to evade the search parties.^14^ Besides such attempts to escape the measures, they staged violent riots and protests against the forcible removal of the sick from homes and the violation of women’s honour in the name of plague searches, both in Bombay and other plague-afflicted parts of the Presidency, like Baroda, Nasik, Poona, and Karachi.^15^ The public’s fury ultimately led to the assassination of the Poona Plague Commissioner, W. C. Rand, who was infamous for his notorious plague measures.^16^

The governmental plague policies underwent a shift from direct interventionism to strategic co-option following Rand’s assassination in 1897. Enlisting selected local elites for the popularisation and implementation of plague measures among the masses, the government advanced sanitary practices and medical procedures, like Prof. Waldemar Haffkine’s anti-plague vaccination, through them.^17^ Additionally, the urban elites contributed significantly to building the city’s medical infrastructure and facilities. When the plague broke out in 1896, there was only one hospital, the Municipal Hospital for Infectious Diseases in Arthur Road (or the Arthur Road Hospital), for the accommodation of plague cases in the city.^18^ A separate hospital, the St. George’s hospital, was exclusively maintained to treat Europeans who contracted the plague.^19^ It was not until December 1896 that the government began acquiring more buildings to be converted into plague hospitals for the Indian residents of the city.^20^ Additionally, to address the public’s objection to the disregard of their caste rules in general plague hospitals, the Plague Commissioner, William Gatacre, exhorted various caste and religious communities to erect new hospitals for treating members of their communities.^21^ Consequently, twenty-nine such hospitals, funded and maintained by prominent religious communities, were built, of which ten were for the Muslims, one for the Parsis, one for the Jews, and seventeen for the Hindus.^22^

Despite the robust body of scholarship on the Bombay plague, aside from a few attempts to portray women’s role in the plague work, much of the existing research portrays the epidemic landscape as a hypermasculine domain, primarily controlled and managed by men.^23^ Two scholars who have attempted to recover the female voices of the time are Ian J. Catanach (2007) and David Arnold (2023). By bringing together the dissenting voices of the British doctor Edith Pechey, of an Indian social reformer, Pandita Ramabai, and of numerous women who nudged their men to protest against the government’s interventionist plague measures, Ian Catanach highlights the women’s labours in the plague years.^24^ Two decades later, David Arnold made a similar attempt to resurrect female presence in plague-stricken Bombay by unveiling the lives and experiences of three British doctors, Alice Corthorn, Margaret Christine, and Marion Hunter, who worked in the plague hospitals in Bombay and its outskirts during the late nineteenth and early twentieth centuries. Being a gender minority in a largely male-driven medical workforce, these women were discriminated against in their workspaces, and their careers in Bombay were short-lived.^25^

Besides reviving these female voices, Arnold’s essay is of particular significance to this paper, for it initiates a brief discussion on the presence of Western nurses tending to the afflicted populace of Bombay. Examining the official correspondence of British doctors and plague officials, Arnold notes that the British men in Bombay were divided in their opinions about the importation of Western nurses from England. While some like Dr Lloyd Jones lauded their work in the plague hospitals, many other British medical officers, he notes, considered these nurses as mere aesthetic projections of the empire, whose ‘presences were glorious, but it was not nursing’.^26^ Given their lack of experience living in a colony and their ignorance of the local languages, these nurses, they opined, could offer no significant contributions to the medical work in plague-run Bombay. Furthermore, Arnold highlights the racial bias inherent in Western nurses against the Indian nurses, popularly known as the ward ayahs, who were only deemed good enough to carry out menial chores in the wards, like cleaning after the patients or changing their bedpans. The British colonial nursing in Bombay, Arnold notes, thus needed an ‘underclass of the native ward ayahs’ for its day-to-day functioning.^27^ Although Arnold’s work offers an entry point into discussing non-military nursing during the Bombay plague years, given the governmental nature of his sources, it fails to record the Western nurses’ interactions with the populace. Their experiences of kinship with the colonised and their discontentment towards the empire go unnoticed. This paper attempts to nuance the relationship between the colonial nurse, the population she tended to, and the empire she served by studying their representation in two different genres – the popular periodical publications of the time and the private letter correspondences of a nurse who worked in Bombay.

## Colonial nursing in British India

Nursing in colonial India had a rather fragmented beginning in the nineteenth century. The colonial state, argues Madelaine Healey, readily and persistently tolerated an underdeveloped nursing system, engaging only minimally with the concerns of the nurses in India until the late 1930s.^28^ Hence, most of the primary initiatives for the growth and evolution of the profession were driven by dedicated individuals, social organisations, philanthropic efforts, and missionary work. Early attempts to bring medical aid and nursing services to India focused on catering to the European community. In the first half of the nineteenth century, British women in India provided nursing care and midwifery services to British communities throughout the subcontinent.^29^ Some of these women also extended their medical aid to the ailing Indians, especially towards the Indian women. Referring to themselves as ‘doctresses’ or ‘lady doctors’, these women engaged in amateur medical practice amongst the colonised, equipped with medical guides, kits, tinctures, and tools that they had brought from England to sustain themselves in the tropics.^30^

A renewed interest in colonial nursing in India was sparked by the Indian Rebellion of 1857, when the casualties of the event exposed the lack of adequate medical aid and good nursing for the British in India.^31^ Notable efforts from British women, like Lady Canning, the wife of Governor-General Charles John Canning, and Sophia Cotton, the wife of Bishop George Cotton of Calcutta, alongside other leading British women, led to the founding of the Calcutta Hospital Nurses Institution in 1859.^32^ It was a private charity that provided nurses for the European wards of the Medical College Hospital and the Presidency General Hospital, in addition to supplying private nursing services for the elite Europeans of the Presidency for a fee.^33^ In 1863, Florence Nightingale, through the Report of the Royal Sanitary Commission of Bengal, suggested that trained nurses be recruited from England to care for the wounded soldiers in India. It was only in 1888, with the arrival of ten trained nurses in Bombay, that the Indian Nursing Service, or the INS, was officially launched.^34^ These nurses were under the supervision of Nurse Catherine Grace Loch, who later protested against their biased treatment by the Army officers and male doctors in the Indian military hospitals.^35^ Besides these efforts at war nursing, scattered attempts were made by motivated individuals to ensure nursing care for the Europeans in India. A few such individual initiatives were the Up-Country Nursing Association, founded in 1892 to provide private nursing care for European residents on a subscription basis, the Lady Ampthill Nursing Institute, founded in 1904 in Madras, and the Lady Minto Nursing Association, established in 1906.^36^ Finally, in an attempt to lay down a common code of conduct for the vocation and to set rules for uniform training of all nurses in British India, the Trained Nurses Association of India (TNAI) was established in 1908.^37^

Contrary to such active measures to meet the nursing needs of the resident Europeans, nursing amongst the colonised was perceived as an afterthought, as a supplement to recruiting female doctors to India. Women missionaries to India in the nineteenth century were the first to recognise the importance of providing medical aid to the Indians, especially to Indian women. Limited by their purdah observances, the zenanas restricted the entry of male doctors, and therefore, Indian women were denied skilled medical aid. Armed with religion and medicine, women missionaries secured entry into the previously inaccessible private spheres of Indian elite women, the zenanas.^38^ Through their mission reports and personal writings, they roused public feeling towards the neglected conditions of the sick women and children, thus leading to the Zenana mission work in different parts of India.^39^ In 1867, the Society for the Propagation of the Gospel (SPG) established the first female medical mission in India, the Delhi Female Medical Mission. They managed and catered to a large population of women through their rudimentary dispensary and nursing home in Delhi.^40^ Female medical missionaries with a basic training in Western medicine and practice were considered suitable emissaries who could advance the mission’s larger agenda of religious conversion among the colonised, alongside offering medical aid. In fact, the mission hospitals they managed distributed Christian tracts and Bibles to the visiting women patients, and routinely incorporated scripture readings into their medical practice, thereby ensuring that a dose of religion was administered much before treatment commenced.^41^

The first fully qualified female medical mission doctor to arrive in India was Dr Clara Swain, who was recruited to work in India by the American Methodist Episcopal Church in 1869.^42^ In 1880, Fanny Butler, one of the earliest female graduates of the London Medical School, was the first medically qualified British woman missionary to serve in India under the Church of England Zenana Missionary Society.^43^ Additionally, Anglo-American Christian missions, the London Missionary Society, the Christian Missionary Society, American Baptist, and Lutheran missions also recruited fully trained, professional nurses from the West to India.^44^ The arrival of qualified medical women heralded the rise of hospitals as the primary space of medical mission activity, in the aftermath of which a need was felt for training more women in nursing services.^45^ In addition to providing qualified doctors, the missions set up nursing schools in India, focusing on training a local corps of nurses. In 1882, the Sisters of the Community of St. John the Baptist of Clewer trained local nurses and supervised the staff at Eden Hospital in Calcutta.^46^ Many women missionaries associated with Anglo-American Christian missions, the American Arcot Mission, the Christian Medical Association of India, etc., are known to have directed the training of Indian nurses in different parts of India throughout the nineteenth and twentieth centuries.^47^

Apart from women missionaries, many British women and wives of British employees in India were instrumental in conceiving of and executing the recruitment and training of Indian women in medicine. The most notable of them was Lady Dufferin, who, at the bidding of Queen Victoria, constituted the National Association for Supplying Medical Aid to the Women of India in 1885 to support the education and training of Indian nurses. The association also instituted the Countess of Dufferin Fund to cater to the medical education of Indian women and establish hospitals and dispensaries for their care.^48^ Furthermore, a crucial part of nineteenth-century efforts to develop colonial nursing was funded through philanthropy. As early as 1871, Lord Napier of the Madras Presidency, with the help of Florence Nightingale, encouraged nursing and trained nurse probationers in the Presidency.^49^ In the Bombay Presidency, Indian nurses were trained in the private Cama Hospital, founded by Pestonjee Hormusji Cama, under the care of Miss Edith Atkinson, who had worked as the lady superintendent of the hospital since 1886.^50^ Additionally, the Jamshedji Jeejeebhoy Hospital in Bombay started training nurse probationers from 1891.^51^ An Indian woman reformer from Bombay, Ramabai Ranade, also trained nursing candidates in her home, Seva Sadan, as a means for the socially marginalised women to earn a livelihood.^52^

Despite these efforts, the nursing profession did not appeal to the Indian women of the middle or the upper classes. Commonly perceived as ‘polluting work’, which involved interacting with strangers and their bodies, the profession defied all caste and ritual pollution rules of the Indian society.^53^ Moreover, until the 1930s, the majority of Indian candidates for nursing were widows, orphans, or destitute converts, who saw the profession as a foothold to social acceptance. The monopolisation of the profession by such lower-class, minority groups further tarnished its social image.^54^ Additionally, the association of Western nursing with Christianity and its unfolding in India through missionary groups further alienated the Hindu communities from taking up the vocation.^55^ Therefore, the extant community of nurses in India was largely comprised of Anglo-Indian or Eurasian women, who, owing to their mixed-race identities, were regarded by both the Indian and European communities as ‘loose’ women.^56^ Consequently, there was a heightened and persistent moral suspicion attached to the profession, which deterred Indian girls from ‘good families’ from taking up the training. Finally, with the emerging opportunities for Indian women to obtain medical education from schools in India and abroad, the most ambitious of them preferred to train as doctors rather than nurses.^57^ Thus, although many organisations and individuals were focused on training Indian nurses and elevating the social status of the profession, it was not the most sought-after career path amongst women of high standing in society.

Notwithstanding these multi-nodal attempts at advancing the nursing profession in India, a consolidated, government-sanctioned attempt to bring Western nurses to serve the afflicted residents of colonial India appears to have been made only during the Bombay plague outbreak of the late nineteenth century. The enormity of the epidemic and severe criticisms about the government’s mismanagement of the disease in Bombay led to the setting up of governmental plague hospitals and medical camps throughout the Presidency. Although many medical missions and nuns volunteered at these hospitals, a fact we learn from the Plague Photograph Album, a strong need was felt for trained Western nurses to run these hospitals and manage the staff.^58^ Hence, the plague years mark a crucial juncture in the history of colonial nursing in India, for, unlike the case of war nursing, wherein the European nurses tended to their countrymen wounded and maimed by the war, the plague-ravaged Bombay presented an uncommon medical scenario – of having to care for the sick, racial ‘Other’. Furthermore, although there may be overlaps in the experiences of war nurses who served the British soldiers and the civilian nurses who served the populace during the plague, the latter’s history merits a standalone inquiry. They were, I argue, multiply positioned – empowered as well as discriminated against – within the peculiar social and political environments they inhabited, further aggravated by the epidemic.

## Representations of Western nurses in Victorian periodicals: A rhetoric of imperial benevolence

The Bombay plague outbreak was one of the first extensively documented medical crises in British India. The colonial archive comprised administrative reports, letters, photographs, and scientific literature on the governmental plague work in Bombay, but their circulation was limited to the bureaucratic circles. Contrarily, the popular press and periodicals relayed news of the riots and racial conflicts between the government and the Indians to the English reading public in the Metropole. Studying the synergistic relationship between the British press and the Empire in the nineteenth century, Julie Codell argues that the press wrote what may be called ‘imperial co-histories’ wherein Britain and its colonies textually constituted their identities.^59^ Although the British newspapers reported news on the plague, I posit that the contemporary Victorian periodicals offered a relatively more accessible platform for open discussions and debates on the governmental plague policies in Bombay. This was made possible by the peculiar mixed nature of the periodicals, which combined verbal text and images, and allowed for heterogeneity of form and authorial voices.^60^

The Bombay plague was a recurring topic across a range of narrative forms in contemporary periodicals, like the *Pall Mall*, the *Good Words*, the *Longman’s*, *The Saturday Review*, the *Temple Bar*, *The Review of Reviews*, and *Blackwood’s Edinburgh Magazine*, to name a few. They carried political writings, personal interviews, and commentary lauding the tough and selfless work of British officers and soldiers in Bombay. Furthermore, they featured descriptive eyewitness accounts of the horrors of the affliction, and fiction set in plague-stricken Bombay, repeatedly portraying the British as altruistic saviours of the colonised. Together, these periodical articulations of the plague portrayed the British government in Bombay as benevolent rulers of the land, thereby attempting to mitigate the English public’s suspicions over the brewing discontent against British plague policies in Bombay. It is against this background of the role of Victorian periodicals in mediating the discourse on the Bombay plague in the Metropole that I situate this inquiry into the periodical presentations of the Western nurse.

Before examining the periodicals, it may be worth noting that the figure of a nurse had had a literary precedence in nineteenth-century England. The Victorian definition of a nurse evolved from its initial perceptions of a kind, laywoman-turned-care-giver of the 1820s to a well-trained, professional, modern, Anglo-American nurse in the 1850s, following the Crimean War. This model of the modern nurse, exemplified by Florence Nightingale, soon found a seminal place in Victorian literature and society.^61^ The image of the ‘heroic nurses’ she championed, as powerful bearers of social reform and redemption in Victorian society, was a sought-after trope among prominent British novelists.^62^ Victorian England was troubled by the possible subversion of social and gender roles that the image of the self-independent heroic nurse could bring about.^63^ Furthermore, portraying the colonial nurses’ engagements with the colonised people, these novels also reflected the racial tensions inherent in British imperialism.^64^ Interestingly, even though these novels preceded the Bombay plague, the periodical representations of the Western nurse in Bombay reflected neither the aspects of the heroic, modern nurse nor that of the stern, racially biased coloniser. Contrarily, examining selected periodical articles of the time, we see the colonial nurses emerge as shallow characters, persistently echoing the benevolence of the imperial project.

Of the periodical articles that discussed the Bombay plague, I examine two accounts specifically focusing on the Western nurse in the plague hospitals. The first article was published in the *Good Words* periodical in December 1899 under the signature M.A.M. Titled ‘Plague-Stricken Bombay: A Peep into the Plague Hospitals of the City’, it is an eyewitness account of a visit to the city’s plague hospitals.^65^ The narrator, notably a male witness, declares his narrative purpose as ‘to tell how human sympathy is endeavouring to relieve the sufferers, and to give some impression by a brief sketch of a visit to the hospitals of what is being done and suffered’.^66^ Nevertheless, despite being an eyewitness record, the author does not provide any details about the hospital, its location, or the names of the nurses he interviews. Thus, the article presents a certain generic description that may fit any plague hospital and any British nurse working in Bombay during the epidemic. Furthermore, going astray from the professed aim of capturing the scenes of the plague hospital, the account chiefly engages in a panegyric on the sacrificial nature of the Western nurses who served the Indians in the plague hospitals. Describing them as those who had been sent ‘out from home on plague duty’, the narrative affords a disproportionate significance to recording the dire circumstances of the nurses’ plight in Bombay over the dismal state of the masses.^67^

The perspective of the witness-narrator in the article is shaped by a determining male gaze, reflecting the stance of a paternal imperialist. Explaining the notion of male gaze, Laura Mulvey notes that

The woman stands in the patriarchal culture as a signifier for the male other, bound by a symbolic order in which man can live out his phantasies and obsessions through linguistic command by imposing them on the silent image of woman still tied to her place as bearer of meaning, not maker of meaning.^68^

Borrowing Mulvey’s notions, I explore how the male gaze in the article appropriates the female Western nurse for articulating the imperialist agenda. The narrator’s depiction of the Western nurses seldom deviates from their conventional representations as skilled medical agents and altruistic caregivers. This controlled presentation may be understood in the context of a crisis of female sexuality in nineteenth-century colonial nursing. Imperial nursing enterprises in several British colonies faced censure over their failure to control the sexuality of the nurse recruits. Fearful that these nurses may engage in inappropriate relations with the men in the colonies and bring shame to the Empire, controlled surveillance was practised through a code of conduct for their dressing and behaviour in the colonies.^69^ A similar sense of control emanates from the portrayal of nurses as de-sexualised beings in this eyewitness account of them. The male narrator upholds their professional abilities as he writes, ‘they were full of knowledge about the diseases and the patients. When showing each patient, the nurses described their illnesses, physical conditions, and recovery statuses’.^70^ Further alluding to their emotional strain from encountering numerous deaths around them, the narrator quotes one of the nurses as saying, ‘Sometimes we think a patient is recovering and we leave him for five minutes. On our return, we find him dead’.^71^ Additionally, by recounting their hardships during the monsoon months, working through the heavy rains due to the poor infrastructure of the hospitals, the narrator constructs them as mirror images of the ‘kind and selfless empire’ in Bombay.^72^ Thus, oscillating between being adept caregivers and exemplars of altruism, the narrator-witness confines the depiction of the nurses within the boundaries of empathy and sacrificial femininity.

Moreover, showcasing the nurses’ interactions with Indian patients, the narrator fashions a seemingly impartial presentation of the races in the plague-run colony – as peacefully coexisting and mutually enabling groups. Consider the presentation of the convalescent wards, which the author describes as ‘full of good cheer, gratitude and self-satisfaction’. He adds how the convalescents who leave the hospital are ‘full of praise for the kind treatment’ they received in the hospital. ‘They are truly grateful for the unselfish care which has been bestowed upon them’. Quoting the nurses’ opinions on the outcome of their work, he adds, ‘Here in the hospital, they have comfortable cots, a well-ventilated ward, and kindly English nurses to tend them, and they are satisfied’.^73^ Here, the article impresses upon the English reader that the poor and desperate Indians need an English nurse to help them survive the epidemic. Underscoring this claim, he adds how nothing could surpass the ‘patience of the suffering people and the devotion to sacred duty’ of the ‘noble and self-denying nurses’ who minister to them. Thus, the article juxtaposes the ‘intensest suffering’ of the patients and the ‘evident signs of kindly aid’ that the author witnessed in the ‘unceasing care’ of the nurses in the plague wards.^74^ Presented thus, the male gaze projects the imperial desire for prolonged rule through the nurses’ work in Bombay. Their demeanour and approaches to the populace are offered as justification for the British presence in Bombay. Further, overvaluing these nurses as magnanimous, kind, and efficient caregivers, the narrative steers towards concretising the notion of a ‘benevolent’ empire.

The portrayal of the nurses as selfless caregivers, I suggest, stems from the imaginative possibilities offered by the relative absence of British nurses from the news and official plague records. Although there are visual references to the nurses’ duties amidst the sick in plague hospitals in the Plague Photograph Album compiled by the British plague officials, given the restricted circulation of these photographs, the English reading public could not have beheld a collective visual representation of the British nurse in Bombay. It may thus be surmised that the limited visibility of the British nurse and ignorance about the nature of her interactions with the residents of Bombay provided a plain canvas from which the periodical writers could create and mould the character of the Western nurse in Bombay. Therefore, it is not surprising that these articles used her as the rhetorical subject and the ideological ground on which the burden of justifying the imperial work in epidemic-ridden Bombay was placed. Notably, the issues faced by these women nurses in their workspaces and their interactions with their male colleagues, which we see emerge through their self-narrations, do not find an expression in this periodical account.

A second periodical plague article that showcases the life and work of a Western nurse in Bombay is titled ‘Fighting the Pestilence: A Nurse’s Experiences in India’. Published in the periodical *The Leisure Hour* in August 1899, it is a first-person account of a nurse who was recruited from England to serve the plague-afflicted populace in the Bombay Presidency.^75^ Although the article is authored by A. V. Stewart, whether or not it is the real name of the nurse-narrator is debatable. Therefore, I propose to read this periodical article as a fictionalised, semi-autobiographical narrative. We learn from the account that the nurse-narrator was a professionally trained nurse, who had previously worked at a London hospital. In Bombay, she was placed in charge of the government plague hospital in Surat, a city in Bombay Presidency, alongside another nurse, whom she refers to as Sister A. Though religious nuns, popularly addressed as sisters, often volunteered to do care work in Indian hospitals in the nineteenth century, the companion nurse, Sister A, is not one such missionary nurse. This we may ascertain from a reference to her as a ‘London Sister’ who journeyed with the narrator, as part of the delegation sent by the India Office from England.^76^ Furthermore, the narrator uses the term ‘sisters’ as a collective noun to refer to a group of nurses in the article. We see this when she recounts the group’s decision on anti-plague vaccination, noting that ‘four of the sisters decided for and six against’.^77^ Thus, the kinship term ‘sister’ in this context could have implied a shared sense of duty, ‘a cross-cultural continuity of care and commonality of purpose’.^78^

The article begins with the phrase ‘Shiva, the destroyer has come!’, reiterating the Indian belief that the fury of the Hindu God Shiva has caused the onset of the plague.^79^ The nurse’s avowal of the medico-religious nexus to define the plague is significant, for it helps obscure the longstanding critique in the Metropole of the British government’s disregard for the populace’s traditional medical beliefs that diseases were curses sent down by their displeased deities.^80^ Therefore, with the invocation of the local deity, the nurse sets herself as an ‘empathetic’ narrator, willing to understand the dynamics of the disease from an Indian perspective. Towards this, the nurse-narrator adopts the rhetorical style of sentimental Victorian travel narratives, wherein her narrative authority lies in the authenticity of somebody’s (her or the populace she writes about) felt experience.^81^ Further, following the mode of sentimentality, her portrayal of the colonised does not take the form of vicious savages, but she casts them within a new sentimental stereotype as ‘benign, ingenuous, child-like victims’, dependent on the coloniser.^82^ Moreover, women travel writers, it is argued, occupied a split position as travel narrators, owing to their racial superiority in the colony and gender inferiority as female colonisers. Their ambivalent position allowed them greater access and insights into those aspects of Indian lives that were off limits for the British men in India.^83^ Although not a traveller, the nurse-narrator, too, had privileged access to the cultural and religious belief systems of the people, owing to her profession. Wielding the cure of medicine during an epidemic, the nurse-narrator secures the trust of the populace she brings relief to. The narrative, thus, features a seamless weaving together of a story of medical salvation with objective descriptions about the land and its culture.

The nurse-narrator claims credibility as a nonpartisan witness through descriptions of the land and the people, evoking the travel narrative paradigm. For instance, she begins her account with a geographical description of Surat’s location in Western India.^84^ Further along, describing her first impressions of India, she writes:

Today, for the first time, I saw India, ‘the land of beauty and mystery’…The jaunty yellow hats of the native police struck us in marked contrast with the enormous sun-helmets of the European officials, while the conical ‘erection’ of the Parsi made the gorgeous turban of Hindu and Mussulman appear all the more brilliant and picturesque.^85^

Resembling an ‘arrival scene’ in a traveller’s account, which sets the relationship of the colonial traveller to the land, the nurse-narrator’s description sets the tone for her ‘production of an “Other” who negates her “Self”, as completely other’.^86^ Although appreciative of the ‘beauty’ of the land and the ‘gorgeous’, ‘brilliant’, and ‘picturesque’ scenes she witnessed, she swiftly exoticises both the land and its people, marking their ‘mystery’ as different from her race.

The narrator maintains this racial difference throughout the account, for it sustains the imperial enterprise of the colonial ‘Self’ improving the inferior ‘Other’. Nurturing, caring, and emotionally invested in the miserable conditions of the colonised, the nurse-narrator, I note, appears to be what Barbara Ramusack calls a maternal imperialist.^87^ Believing that they belong to a superior civilisation, which imbues them with the ‘duty’ to bring ‘civilisation’ to the savage parts of the world, the maternal imperialists cast themselves as ‘mothers’ to their ‘unruly’ children of the colonies. However, the problem with this fictive kinship, argues Ramusack, is that it is founded on an unequal racial relationship – mothers are always European while the Indians continue to be infantilised as their dependent wards.^88^ A similar sense of inherent superiority and maternal affection runs through the nurse-narrator’s account of her experiences in Bombay and interactions with the Indians. Moreover, her maternal imperialist stance is powerfully conveyed through the mode of self-narration, which lends the account an apparent tone of candour, as opposed to the third-person, eyewitness’s narration in the previous periodical article.

Portraying themselves (Sister A and herself) as people who left behind the comforts of their home country to serve the needy, the author elevates the profession of nursing as a selfless devotion. Extrapolating this stance, she interweaves discipline and routine alongside empathy and compassion to define the work of a nurse. Summing up her day’s work, she writes:

I used to rise before daybreak, so as to get over to the hospital early, and then the work of the day began with counting the dead… The wards cleared of the dead, cleaned and aired, we had a busy time with taking the temperature, pulse etc., and getting the patients ready for the early visit of the doctors. Medicines, fresh dressings to the swellings or buboes, the supervision of the distribution of food to the patients and their friends, made the time fly till the daily official round of the civil surgeon.^89^

The narrator further elaborates the mental and emotional exertion of their work, from having to encounter countless deaths in the wards every day; ‘It was literally work among the dead and dying. The entrances and passages were often full of patients whose beds there was no room in the wards till the dead had been carried out’.^90^ She further recollects the case of a little boy orphaned by the plague: ‘It was a strange and pathetic sight to see this tiny survivor of a whole family, sitting on the floor amidst the dead, who had doubtless loved him so dearly, with childish ignorance clamouring for the bowl of milk which Tukeram, my old ward-boy, was feeding him’.^91^ Noting how she cared for the boy, bathed, clothed him, and found him a good family to live with, she remarks how the Indians anticipated that she would adopt the little boy. All her declarations of selfless devotion and duty described above carry an implicit assertion of her colonial authority, as she reiterates that it was her timely intervention in their miserable lives that offered them salvation.

The nurse-narrator’s focalisation of a sentimental traveller is, at times, supplanted by an ethnographic gaze. Combining elaborate descriptions of the cultural practices of the populace with visual illustrations, her ethnographic persona is underscored by her professed attempts to comprehend the cultural and religious sentiments of the people she cared for. Elaborating on a curious Indian custom of keeping a lamp lit on the floor and constantly burning beside a dying person as his last breath leaves his body, she remarks:

To our Western minds, the appearance of the wards at break of day was too awful. Here some unfortunate had evidently died in the act of trying to reach the ground. There another had fallen in a heap in the same attempt, while empty beds with corpses by the side showed where the faithful friend had carried out the last wish. With the first light of the morning, the relations trooped in to carry away the loved ones, and I could not but be touched with the reverence and refinement which accompanied the last offices.^92^

Further, describing the preparations for cremation, she writes,

Two women held a sheet as a screen, whilst the nearest relation washed the body and wrapped it in a red or white cloth. The corpse was then strewn with flowers, placed on a bamboo bier, and carried to the banks of the Tapti, where it was dipped in the sacred waters, and finally burnt.^93^

**Figure 1.**
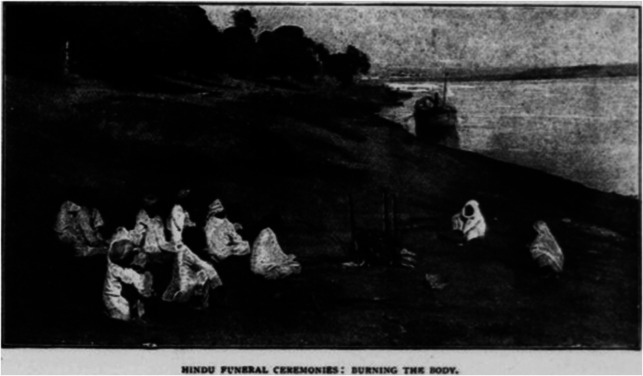
Depiction of cremation, with the body burning on a pyre and the mourners seated around it. From A.V. Stewart. ‘Fighting the Pestilence: A Nurse’s Experiences in India’. *The Leisure Hour*, August 1899 (Source: ProQuest Database, accessed on 13 August 2020)

**Figure 2.**
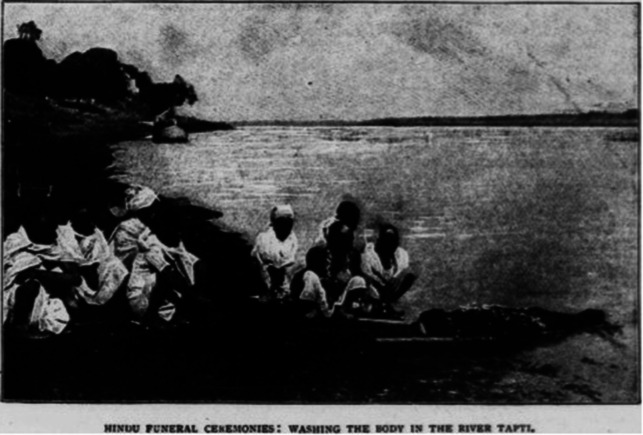
An image showing a corpse being washed on the banks of the Tapti River before cremation. From A.V. Stewart. ‘Fighting the Pestilence: A Nurse’s Experiences in India’. *The Leisure Hour*, August 1899 (Source: ProQuest Database, accessed on 13 August 2020)

These detailed descriptions were accompanied by a visual record of the death rites and cremation pyres (Figures 1 and 2).

The photographs in the article are similar to the ones in the government’s Plague Photograph Album. Despite the nurse’s professed claims about her desire to ‘comprehend’ the Indian culture empathetically, the presentation of these images, I note, is not without its politics. Citing the profusion of images of corpses and cremation pyres in the Plague Photograph album and sketches of the blazing funeral pyres in illustrated newspapers of the time, David Arnold argues that these images were used to emphasise the primitivism of the Hindu cremation.^94^ These representations generated an unsettling (yet alluring) spectacle of ‘oriental’ exoticism and marked the bizarre ‘eastern’ bodily practices and ‘stranger’ funeral rites as far removed from the Victorian conventions of death and burial.^95^ Building on Arnold’s observations, it may be surmised that the images of cremation from colonial India were a curious spectacle that had an audience in the Metropole. Moreover, these images of cremation rites showcase and underscore the difference between the ‘primitive’ Indians and the people of the Metropole. The inclusion of these visuals in the nurse’s account underscores the cultural estrangement faced by the British in India, thus impressing on the minds of the English reader an image of an empire inclined to ‘know’ the Indian ways of life and death, while planning the anti-plague measures. The portrayal of these Indian customs, when viewed in the light of the criticisms against the governmental plague measures’ insensitivity towards the populace’s religious and cultural sentiments, may be read as a calculated narrative strategy to gesture towards the Empire’s humanitarianism.

Finally, digressing from her role as a medical saviour, the nurse-narrator draws on anecdotes from her workspace to imply that the Indians she tended to welcomed and valued her presence in their lives. Most of her anecdotes feature recently orphaned or abandoned children of the plague victims. One such episode she recollects is of a five-year-old boy who had accompanied his sick mother to the hospital. The boy soon turned to be her constant companion, ‘trotting up and down after me and insisting, in spare moments, on fun and frolic’.^96^ Another twelve-year-old girl, who had lost her parents and siblings, she recollects, nursed her only surviving three-year-old brother to health. Describing her departure from the hospital, she writes, ‘It was a grand day for the little heroine when she carried her brother out of the hospital, both resplendent in the gorgeous new clothes we provided for the occasion’.^97^ As may be noted in these two anecdotes, all of her interactions with the Indians end on a certain note of kindness she bestowed upon them. Implying a sense of parity and reciprocity in her engagements with the Indians, the nurse-narrator concludes her account by advocating for a mutually enabling relationship between the two races. She adds:

I cannot but think that the experiences of this terrible pestilence will make a wider breach in the wall of antagonism between ruler and ruled, and that, with the entry of the fresher air of knowledge and understanding, in the years to come the native may realise that mercy and justice move hand in hand with the government of the British Raj.^98^

Though the ending may, as in the previous periodical article, give the reader a sense of a balanced portrayal, the mutual relationship she urges through her account tacitly promotes the unopposed continuance of British rule in India, so that the ‘needy and confused’ Indian may be helped and supported in critical times like the plague. Finally, by portraying the relationship between an English nurse and an Indian patient as agreeable to one another, the process of British imperialism is ‘camouflaged’ as a desirable design that benefited the governed much more than the rulers.

Although mediated through different focal points – of a male eyewitness’s gaze in the descriptive account and a maternal imperialist’s perspective in the semi-autobiographical account of the nurse-narrator – the two periodical plague accounts reveal a shared rhetoric of carefully managed presentations of the Western nurse in Bombay. Portrayed as models of unreasonable altruism, incredibly sympathetic and forgiving, and as displaying excessive maternal affection towards their patients, they are a far cry from the Victorian new heroic nurse, who is a professional woman of distinct individuality. Unlike their complex and often subversive portrayals in the novels as possible threats to the social and gender order in England, the nurses in these narratives are presented as complicit in the imperial project and as complementing the patriarchal order within the British Empire. As such, the figure of the nurse in the periodical plague narratives functions as a synecdoche for the empire, always speaking on behalf of the empire, and shielding British rule in the colonies from its critics in the Metropole.

## Autobiographical nurse as an ambivalent subject

In her essay ‘A Woman’s Trek: What Difference Does Gender Make?’, Susan L. Blake explores how a woman’s narrative perspective alters the descriptions of the colonised and the imperial ideology in European travel accounts from the colonies.^99^ Comparing the travel account of a woman traveller, Mary Hall, to colonial Africa against two contemporary male travellers’ accounts from Africa, Blake contends that, ‘A woman’s point-of-view does not guarantee a reciprocal relationship with an Other, but it opens a crack in the concept of Self through which to examine the concept of “Other”’.^100^ Building on Blake’s argument that gender opens up new entry points to understand the ‘Self-Other’ dynamics in a colonial setting, I explore how the intersecting identity politics of race, class, and culture further nuance the figure of a Western nurse serving Indians in Bombay. Examining an altogether different form from the periodicals, this section analyses the personal letters of a British nurse, Hester May Dowson, who served in Bombay from 1897 to 1898.^101^ Attending to the distinct form and expressive possibilities allowed by letter-writing, this analysis also demonstrates how colonial women’s standpoints were not only shaped by their positionalities but also by the nature of the narrative forms that they adopted to articulate their experiences.

Hester May Dowson was one of the many British nurses recruited for plague work in Bombay. Dowson and her friend, Nurse Harriet McDougall, worked in the Parel Governmental Plague Hospital from 1897 to 1898. Selected extracts from her letters from Bombay to London were posthumously compiled as *Bombay During the Plague, 1897–98: Extracts from the Letters of H.M.D.* Contrary to the periodicals’ defined public readership in the Metropole, Dowson’s letters were composed to her relations in London. Thus, the privacy, confidentiality, and familiarity of recipients of the epistolary medium provided Dowson an opportunity to pen her genuine thoughts about the people, the plague, and the government’s plague policies. The image of the nurse that emerges through Dowson’s letters cannot be neatly classified as either complicit with or in opposition to the British government’s plague policies. Situating her letters within discourses on nineteenth-century British women’s intimate letter writing and the rhetoric of British women’s writings from colonial India, I contend that the non-military nurse, caring for the colonised Other during a medical crisis, occupied an ambivalent position that opened up newer perspectives on colonial relations.

Writing in 1898, Nurse Dowson’s letters follow the tradition of nineteenth-century letter writing, especially by women from different parts of the empire. Women, particularly migrant women, wrote back to their families, friends, or their spouses detailing their lives, work, routine, and fears, as a way of maintaining their familial links, rehearsing or creating familiar stories, and retaining a sense of shared identity, while settling in new and distant places.^102^ Examining many such letters, Monagle et al. argue that nineteenth-century women’s letters differed from those of earlier periods through the notion of intimacy they contained.^103^ In the context of women’s letter-writing, the essence of the term ‘intimacy’ lies in the confidential nature of the form made possible by the renewed notion of the ‘addressee’, which had by then acquired the meaning of being the only one (or ones) who read the contents of the letters. Assured of a confidential dialogue with the addressee, this marked a shift from the old practice of reading letters aloud to a wider, unfamiliar audience.^104^ Resorting to informal language and expression of feelings, these letters enabled them to recast their sense of self through a new kind of frankness and self-disclosure, often impossible to incorporate into conversations.^105^ Thus, these women writers could carve out letter writing as a ‘private’ act.^106^

Hester Dowson’s informal style and candid tone of writing suggest that, like her contemporaries, she too shaped her epistolary persona, reassured by the intimacy and privacy afforded by the letter form.^107^ Although the book cover does not carry publication or editorial details, it is mentioned that it was meant solely for private circulation. Similarly, being a compilation of fragments of her letters, information regarding the addressee(s), her relationship with them, the typical length and frequency of the letters, and the exact duration of the correspondence from Bombay cannot be reliably ascertained. Nevertheless, from the available sequence of letters, they seem to have been sent twice a week. Additionally, putting together occasional references to her interlocutors, her letters seem to be addressed to a familiar, collective audience of her family, and were probably read aloud to them.^108^ Her familiarity with the addressee(s) may be understood from her use of collective referential pronouns and words of endearment when she writes, ‘Over and over again, I think it is almost worthwhile coming away to get such dear letters and sweet thoughts from you all, and to find out what a great deal we care about each other, though we are seldom much together’.^109^ Additionally, the majority of her letters note how much she missed them and longed to see them. Notably, she does not seek counsel from them or display excessive deference in her letters. Rather, her epistolary persona comes across as a confident young woman, certain of her life choices and informing her kin back home of the ingenious ways in which she managed her difficult life in a faraway land, suggesting she shared a close relationship of relative equality with them.

Dowson’s letters largely focus on adjusting to life in Bombay as a colonial nurse working amongst the Indians. Just like the nurse figures in the periodicals, she narrates the trials of her job while tending to the afflicted people. However, her sufferings and sacrifices are not the only focus of her letters. The majority of her letters detail the interpersonal relationships she formed in Bombay, including her friendship with her companion nurse, Miss McDougall, her relationships with the British community in Bombay, day-to-day interactions with her servant boy, her Goanese cook, and her rather difficult conversations with the Indian orderlies in the hospital wards. Towards the end, she writes about the death of her companion, Nurse McDougall, and we witness her battling the grief of her death as a lone migrant in a foreign space. Unlike the periodical writings, Dowson’s letters do not seem to navigate intentionally towards a particular rhetoric. In fact, the letters are laced with her opinions about the land, its people, and the British officials on plague duty, often featuring outright censure of the government’s anti-plague policies.

Dowson strongly criticises the British government’s lukewarm responses and inefficient provisions to contain the plague in Bombay. She speaks disapprovingly of the deplorable state of the plague hospitals. She confesses that owing to the lack of resources and facilities, the nurses in Bombay found ‘it most terribly harassing and depressing to be obliged for want of the necessary materials, to allow things to slide, and perhaps only do for the patients a little better than would be done in their own homes’.^110^ Given the mental agony the patients go through in these alien spaces, she opines that they may have been better off at home, spending their last days surrounded by people whom they love. Elaborating upon their mental anguish, she notes how the Indian patients in the hospitals were averse to touch – a concern, argues David Arnold, that contributed to broader resistances to Western medicine in Bombay. Dowson notes that most of them disliked being touched or fed by the British nurses. Alternatively, she notes that they would find a ward boy of the same caste as the patient, through whom food and medicines were administered. Citing these instances as obstacles that her job required her to tackle, and not as racialist judgments, she foregrounds the needless psychological trauma to which the Indians were being subjected in the hospitals, against their will.

The futility of the government’s insistence on forcible hospitalisation is further underscored in Dowson’s opinion that the doctors brought from England were new graduates who lacked the requisite expertise to treat the plague patients. Expressing her disdain for their inept work and racial prejudices against Indians they treated, she writes, ‘It sometimes makes my blood boil to hear of, and see how the personal feelings and little caste and religious prejudices of the people are despised and trodden on, especially by these young men fresh from England, who know nothing of the language or the natives’.^111^ Furthermore, describing their reckless treatments, she notes that, to the detriment of the poor patients, they prescribed the same treatment, applying ice poultices to all, irrespective of the specificity of the cases. Explaining the impracticality of such a remedy for the hot and humid climate of Bombay, she writes:

The treatment this Doctor goes in for, is ice to the swellings and any amount of medicine, sometimes 2 hourly, sometimes half-hourly, and about 8 different kinds. Then he has about half the patients on jacket poultices for pneumonia, or to prevent getting it. Of course, in this heat, the air is always condensing on the ice bags, and the consequence is, the patients are often lying in pools of water. We can never hardly have sheets enough to change oftener than twice in 24 hours; and these Indians seem to feel cold all over, and shiver if any ice comes near them, and many of them, I am sure, think we are trying to kill them when we put them in wet packs.^112^

Thus, pointing out the flaws in the medical services offered to the colonised, the nurse’s letters expose the facade of Western medicine that was often upheld as a mark of imperial benevolence. Such expressions of vacuity of the medical enterprise and its imperial agents are absent in the periodical literature, whose narratives are singularly fixated on glorifying the work of the empire in colonial Bombay.

Nurse Dowson’s letters are not just documents exposing the British government’s perfunctory plague policies; rather, they also unravel the government’s discriminatory attitudes towards the female nurses in the colonies. She contrasts their living conditions with those of the male doctors from Britain, who were given better accommodation, additional allowances, and considerably higher pay in Bombay. Evaluating her position vis-à-vis the British male doctors in Bombay, she writes:

I think the government have treated the nurses very badly, our pay is poor for the accommodation and work; they sent us cheaper than 2nd class, down at the bottom of the ship, where it made us all ill if we slept there, the air was so stifling; whereas the Doctors got twice as much for outfit, 1st class return tickets instead of 2nd class single, so that they can return any time, and considerably higher pay out here. One poor nurse they have sent to Surat, a native town where there are hardly any Europeans. She writes me to say her quarters and everything are appalling and she would give all she possesses to get away and yet she had knocked about the world a great deal, in Egypt and elsewhere.^113^

Dowson’s revelation of the gendered nature of the colonial medical realm could be read in tandem with the ongoing resentment against the hierarchical, masculine space of the nineteenth-century British medical realm. Although women doctors from England preferred to migrate to the colonies to advance their medical careers, such gendered biases continued in colonial India.^114^ Dr Marion Hunter, who was the only female doctor to have served in the Poona plague hospital, notes that she was often discriminated against by her male colleagues for being a woman in a medical realm otherwise manned by them.^115^ Similar experiences were revealed by the British war nurse, Catharine Grace Loch, in the memoir of her life and work in the INS (Indian Nursing Service) from 1888 to 1902. Evaluating her circumstances, Loch writes that war nurses, for lack of a rank in the Indian military medical system, were slighted by the orderlies who reported to them as well as insulted by the doctors they worked with, all because the government left all ‘women things vague and unbusinesslike’.^116^ One hears similar echoes of the women medical professionals’ plight in Dowson’s letters.

Dowson’s tone of fearless critique of the British government was not a commonplace occurrence in nineteenth-century British women’s writings from India. Examining the vast body of journals, memoirs, letters, fiction, and sketches of the Memsahibs and women visitors to India from the nineteenth century, Sara Suleri observes that, relegated to the peripheries of colonisation, their writings were veiled behind the trope of ‘feminine picturesque’. They could write about and capture images of people and places from their peripheral vantage points, provided they ‘remained immune to the sociological conclusions of their own data’. That is, they could enter the political domain to aestheticise through the picturesque presentations but not to analyse.^117^ Contrarily, Dowson’s letters enter and critique the political domain of the Empire, questioning the very necessity of British interventions into the Indian medical realm. Her narrative authority, I posit, was shaped by her complex subject position in colonial Bombay. Dowson was unlike the wives of British officers, women missionaries, and female travellers to India in the nineteenth century, who were, argues Ramusack, cultural missionaries and maternal imperialists. Having internalised a Eurocentric ideology, these women’s interactions with the colonised were premised on a self-imposed burden of improving the Other, by bringing hygiene, education, Christianity, and Western morality to Indian homes.^118^ In fact, furthering Ramusack’s argument, Nancy L. Paxton notes that their professed complicity with the imperial enterprise in their writings is a direct reflection of the Victorian morality, sexual discretion, and domestic roles that tied these women to their social and class standing.^119^ Dowson, belonging to the working class of British women in India, did not shoulder the burden of imperial motherhood as these women.

Nevertheless, Hester Dowson’s letters were not entirely free of such racial biases. We see attitudes of Eurocentrism surface when she makes generalisations about the Indian ward boys and the Bombay-trained Eurasian nurses. Writing about her trouble with the ward boys in the hospital, she says, ‘We find it all rather trying to the temper, especially when they will stop and talk and explain in an unknown language, instead of doing work which is so obviously waiting to be done’.^120^ A similar bitterness comes through her observations about the Indian nurses, whose caste she frames as a factor limiting their efficiency at work. Subscribing to the prevalent assumption that the Eurasian nurses were ‘loose women’, she observes, ‘The Bombay nurses are well-trained, but often half-caste or Eurasian, and not so capable at their work as we, nor can they keep order or make the ward boys work nearly as well’.^121^

Notwithstanding her sense of racial superiority, building on Blake’s idea of the gendered nature of colonial travel writing, I argue that Dowson’s letters create a fissure in the imperial fabric. Bound by the nature of her job, she engages closely with the Indian populace and their resistance to Western medicine. Her sustained interactions with the colonised Other helped her reconfigure her imperial Self. Dowson notes in her letters that the agitated patients attacked nurses and pelted stones at them. Defending their actions, she instantly explains that the Indians perceived the nurses as the faces of the Western medical system and held them responsible for the forcible hospitalisation and administration of medicines to them. She writes, ‘… I am afraid, a good many of the natives hate us, and all connected with this plague business’.^122^ Nevertheless, rather than being quick to judge them, putting herself in their shoes, Dowson analyses the impact of their plague measures on the miserable Indians. She writes:

There are a great many native soldiers in Bombay just now, as they are afraid of rioting, owing to the very strict house-to-house visitation that is now going forward, in fruitless hope of stopping the ravages of the plague. And I really should not be surprised if the natives did rise; I think it is awfully hard on them, all these regulations being enforced, simply because we have the power. I am sure most of them do not understand or believe that it is done from a disinterested motive or for their good.^123^

As is evident in the above excerpt, the nurse’s cognisance of the unfair power that imperialism granted her and her countrymen in Bombay prompts self-reflection and introspection. Her letters are suggestive of an alternative intimacy and empathy with the colonised Other that the writings of British women who preceded her fail to achieve. This could be because of her complex subject position: racially superior to the Indians she cares for, yet subordinate within the British colonial hierarchy due to her gender and class. Unlike the Memsahibs and the mission women, she is not bound by marital or social ties. At the same time, she exercises a distinct form of power through her medical expertise and relative financial independence. Further, her ambivalent social and financial position offers her a narrative authority, unavailable to the other women who preceded her.

Situated firmly within her complex subjectivity as an imperial agent and an independent woman, Nurse Dowson’s epistolary persona narratively challenges the uneven hierarchies that construed the colonial ‘Self’ and its ‘Other’. In her letters home, comparing her life in England and India, she expresses an uncommon desire to live in India.

If only you others were here, I should like it better than England, if I could only stop this grinding work, and enjoy it. It is a curiously fascinating and somewhat weird country. So much less prosaic and matter-of-fact than England, and much more peaceful too. I can quite understand there being so much mysticism mixed with grotesqueness in their religions.^124^

Acknowledging differences in the Indians’ understanding of the world, but not condescendingly, she writes:

All out here is quiet and slow; they are all firm believers in ‘kismet’ and have an infinite contempt for the Englishman’s idea of improving things, and ‘helping God’ as they term it, (as if he couldn’t do all these things for Himself if He wished it) but the hindus are a most gentle and affectionate race as a rule, and are more devoted to their wives and children than our people at home.^125^

Describing the land as more peaceful and its people as emotionally and morally superior to the English, the nurse subtly inverts the myth of superior civilisation that held up the imperial enterprise. Unlike the nurse figure in the periodicals, who tried to hide her imperial agenda behind a facade of professed parity and reciprocity between the races, Dowson’s letters subvert the implicit racial hierarchy by elevating India and Indians above the British in the Metropole, thereby opening up a space to reconfigure the ‘Self-Other’ dichotomy.

## Conclusion

Inquiring into the under-researched figure of a trained, non-military nurse from England serving the colonised populace in plague-stricken Bombay, this paper contributes to the extant discourses on women’s labour during the Bombay plague, colonial nursing in nineteenth-century India, and the narrative possibilities furthered by women’s writings from the colonies. Exploring their representations in two different forms, it becomes apparent that the periodical accounts employed the Western nurse as a spokesperson for the government’s plague work, thereby shielding the British rule in Bombay from critics in the Metropole. The two periodical accounts, though differently focalised through the male gaze of an eyewitness account and the sentimental female gaze of a semi-autobiographical account, portrayed a shared rhetoric of the Western nurse as a sympathetic, selfless caregiver.

Contrary to the periodical form, Nurse Dowson’s letters, secured by the intimacy and confidentiality afforded by the epistolary form, offered a nuanced portrayal of the Western nurse’s life and work in Bombay. Highlighting the British government’s perfunctory policies, racial biases towards the colonised, and the gendered attitudes to the female nurses, Nurse Dowson’s letters offered an uncommon critique of the Empire, rarely visible in British women’s writings from the colonies. Moreover, her letters challenged the myth of European superiority by defending the agitated populace’s resistance to the plague policies and arguing that the Indians were morally and emotionally more evolved than the British. Although she occupied the inner realm as an imperial agent, her ambivalent attitude to the Empire may be understood as an outcome of her complex, multi-dimensional subjectivity – racially superior to the Indians she cared for, yet subordinated by gender and class within the British colonial order. Further, unlike the women who preceded her, Dowson was not limited by marital ties or elitist mentalities. Aware of the unequal power dynamics enjoyed by the British in Bombay, her letters showed moments of recognition and self-reflection. Additionally, her medical expertise, close engagements with the colonised Other, and her relative financial stability as a professional nurse conferred on her a distinct narrative authority, which, in turn, fissured the imperial fabric, opening up a space to reconfigure the ‘Self-Other’ dichotomy.

## Footnotes

1

Nupur Chaudhuri and Margaret Strobel (eds), Western Women and Imperialism: Complicity and Resistance (Bloomington: Indiana University Press, 1992), 1–15 .

2
*Ibid.*, 11.

3

Helen Callaway and Dorothy O. Helly , ‘Crusader for Empire: Flora Shaw/Lady Lugard’ in Nupur Chaudhuri and Margaret Strobel (eds.), Western Women and Imperialism: Complicity and Resistance (Bloomington: Indiana University Press, 1992), 79–97 .

4

Charlotte Dale , ‘The social exploits and behaviour of nurses during the Anglo-Boer War, 1899–1902’. in Helen Sweet and Sue Hawkins (eds.), Colonial Caring: A History of Colonial and Post-colonial Nursing (Manchester: Manchester University Press, 2015), 60–83 .

5

Lynda Bryder . ‘“They do what you wish; they like you; you the good nurse!”: Colonialism and Native Health nursing in New Zealand, 1900–40’, in Helen Sweet and Sue Hawkins (eds.), Colonial Caring: A History of Colonial and Post-colonial Nursing (Manchester: Manchester University Press, 2015), 84–103 .

6

Angharad Fletcher , ‘Imperial sisters in Hong Kong: disease, conflict and nursing in the British Empire, 1880–1914’, in Helen Sweet and Sue Hawkins (eds.), Colonial Caring: A History of Colonial and Post-colonial Nursing (Manchester: Manchester University Press, 2015), 41–59 .

7

Mary Louise Pratt , Imperial Eyes: Travel Writing and Transculturation (New York: Routledge, 2008), 7. Here I borrow Pratt’s definition of contact zones as ‘social spaces where disparate cultures meet, clash, and grapple with each other, often in highly asymmetrical relations of domination and subordination – such as colonialism and slavery, or their aftermaths as they are lived out across the globe today’.

8 The British operated trade in opium, textiles, spices, indigo and saltpetre from Bombay to the Middle East and Europe. Additionally, many Indian trader communities also engaged in textile, ship-building and spice trades from Bombay. For more on Bombay’s trade ventures, see Sifra Lentin . Mercantile Bombay: A Journey of Trade, Finance and Enterprise (New York: Routledge, 2022).

9 Prasanth Kidambi, ‘An Infection of Locality: Plague, Pythogenesis and the Poor in Bombay, c. 1896-1905’, *Urban History*, 31, 2 (August 2004), 249–67: 252. Kidambi argues that the British government’s approach to the plague had been one of ‘contingent contagionism’, a theory that enabled an effortless association between the plague and the poorer classes of society. Consequently, they could justify the enactment of anti-plague measures impacting the poor alone. For more on plague policies and class politics, see Rajnarayan Chandavarkar , ‘Plague Panic and Epidemic Politics in India, 1896-1914.’ in Terence Ranger and Paul Slack (eds.), Epidemics and Ideas (Melbourne: Cambridge University Press, 1995), 203–41.

10

David Arnold , ‘Touching the Body: Perspectives on the Indian Plague, 1896-1900’, in Ranajith Guha and Gayatri Spivak (eds.), Selected Subaltern Studies (New Delhi: OUP, 1988), 391–425: 395.

11 Natasha Sarkar, ‘Fleas, Faith and Politics: An Anatomy of an Indian Epidemic, 1890-1925’(unpublished PhD thesis: National University of Singapore, 2011), 150–51.

12 Arnold, *op. cit.* (note 10), 396. Indian society, shaped by a hierarchical caste system, followed a stringent system of untouchability, whereby the lower castes were ostracised as ‘impure’ and ‘polluting’ by the dominant caste groups. They were denied education, legal status and human rights, besides being forbidden from public spaces.

13
*Ibid.*, 399.

14

Mridula Ramanna , ‘“We Thought The Government Was Our Maa-Baap!”: State Intervention And Indian Responses To Plague Control Measures, 1896-1920’, in Health Care in Bombay Presidency: 1896–1930 (New Delhi: Primus Books, 2012), 10–38:13.

15
*Ibid.*,16–20.

16
*Ibid.*, 20.

17

W.L. Harvey , Report of the Municipal Commissioner on the Plague in Bombay for the year ending 31st May 1899 (Bombay: Times of India Press, 1901), 43 . Prof. Haffkine was a Russian bacteriologist invited by the British government to prepare an anti-plague vaccine in 1896. He prepared the vaccine at the Grant Medical College, Bombay in 1897.

18

William Forbes Gatacre , Report on the Bubonic Plague in Bombay, 1896-97-Vol 1 (Bombay: Times of India Steam Press, 1897), 3–4 .

19

J.K. Condon , The Bombay Plague: Being a History of the Progress of Plague in the Bombay Presidency from September 1896 to June 1899 (Bombay: Education Society’s Steam Press, 1900), 76 .

20 Gatacre, *op. cit.* (note 18), 24.

21
*Ibid.*, 24.

22 Harvey, *op. cit.* (note 17), 151.

23

Ian J. Catanach . ‘“The Gendered Terrain of Disaster?” India and the Plague, c. 1896-1918’, South Asia: Journal of South Asian Studies, XXX, 2 (August 2007), 241–67: 245.

24
*Ibid.*, 241–67.

25

David Arnold , ‘Dr Hunter’s Plague: Gender, Race and Photography in British India’, Indian Journal of Gender Studies (2023), 1–21: 12–13.

26
*Ibid.*, 14.

27
*Ibid.*, 13–15.

28

Madelaine Healey , Indian Sisters: A History of Nursing and the State, 1907-2007 (UK: Routledge, 2013), 5 .

29

Narin Hassan , Diagnosing Empire: Women, Medical Knowledge and Colonial Mobility (New York: Routledge, 2011), 3 .

30
*Ibid.*, 3.

31

Sarah A. Tooley , The History of Nursing in the British Empire (London: S. H. Bousfield & Co. Ltd., 1906), 339 .

32 Tooley, o*p. cit.* (note 31), 339; Sneha Sanyal , ‘Emergence of Nursing as a Profession in Nineteenth-century Bengal’, Social Scientist,45, 3 (2017), 69–86: 73.

33 Sanyal, *op. cit.* (note 32), 73.

34 Healey, *op. cit.* (note 28), 70.

35
*Ibid.*, 70; Preethi Mariam George and John Bosco Lourdusamy . ‘Trained Army Nurses in Colonial India: Early Experiences and Challenges’. Medical History,67, 4 (2023), 347–64: 35137828845 10.1017/mdh.2023.31PMC10616692.

36 Tooley, *op. cit.* (note 31), 348–9; Preethi Mariam George , ‘Institutionalisation and Professionalisation of Nursing in Colonial India’, Proceedings of the Indian History Congress, 81 (2022), 738–46: 739.

37 George, o*p. cit.* (note 36), 741.

38

Rosemary Fitzgerald . ‘A “Peculiar and Exceptional Measure”: The Call for Women Medical Missionaries for India in the Later Nineteenth Century’ in Robert A Bickers and Rosemary Seton (eds), Missionary Encounters: Sources and Issues (Great Britain: Curzon Press, 1996), 174–96: 176.

39
*Ibid.*, 181.

40
*Ibid.*, 187.

41
*Ibid.*, 191.

42
*Ibid.*, 185.

43
*Ibid.*, 192.

44 George, o*p. cit.* (note 36), 740.

45 Fitzgerald, *op. cit.* (note 38), 192.

46 George, o*p. cit.* (note 36), 739.

47
*Ibid.*, 740.

48

Maneesha Lal , ‘The Politics of Gender and Medicine in Colonial India: The Countess of Dufferin’s Fund, 1885-1888’, Bulletin of the History of Medicine, 68, 1 (1994), 29–66: 358173304 . Despite their claims, the Dufferin scheme was primarily focused on eliminating the local midwives, at whose expense the British women doctors hoped to secure their medical careers in India.

49 George and Lourdusamy, *op. cit.* (note 35), 349.

50 Healey, *op. cit.* (note 28), 71.

51
*Ibid.*, 72.

52
*Ibid.*, 72-3.

53
*Ibid., 50.*

54
*Ibid.*, 51.

55
*Ibid.*, 13.

56
*Ibid.*, 56.

57
*Ibid.*, 57.

58

David Arnold , ‘Picturing plague: Photography, pestilence and cremation in late nineteenth- and early twentieth-century India’, in Christos Lynteris (ed.), Plague Image and Imagination from Medieval to Modern Times (Switzerland: Palgrave Macmillan, 2021), 128 . The Bombay Plague Photograph Album was compiled in 1897 to document the work of the Bombay Plague Committee, headed by General Gatacre. Although the majority of the photographs are attributed to Captain C. Moss of the Gloucestershire Regiment and F. B. Stewart of Poona, recent research notes an Indian correspondent to *Times of India*, Khemji Hirji Kayani, as being one of its contributors.

59

Julie F. Codell , Imperial Co-Histories: National Identities and the British and Colonial Press (UK: Rosemont Publishing and Printing Corp., 2003), 16 .

60

Margaret Beetham , ‘Open and closed: The periodical as a publishing genre.’ Victorian Periodicals Review 22, 3 (1989), 96–100: 96.

61

Catherine Judd , Bedside Seductions: Nursing and the Victorian Imagination, 1830-1880. (New York: St. Martin’s Press, 1998), 2.

62
*Ibid.*, 22.

63
*Ibid.*, 26. The character of Georgia in Hilda Gregg’s novel *Peace with Honour* (1897) is one such heroic British nurse, whose commitment to the nursing profession signifies an avowal of the ‘new womanliness’.

64

Kristine Swenson , Medical Women and Victorian Fiction (Columbia: University of Missouri Press, 2005), 177 . Rudyard Kipling’s novel *Naulakha: A Story of West and East* (1892), set in colonial India, laid out the racial tensions through the protagonist of the American nurse Kate, who assumed a self-labelled superiority – both intellectual and racial – over the Indian women and their traditional medical knowledge.

65 M.A.M., ‘Plague-Stricken Bombay: A Peep into the Plague Hospitals of the City’. *Good Words* (December 1899), 842–44.

66
*Ibid.*, 843.

67
*Ibid.*, 843.

68

Laura Mulvey , ‘Visual pleasure and narrative cinema’, in Constance Penley (ed.), Feminism and Film Theory (New York: Routledge, 1988), 803–16: 804.

69

Dea Birkett , ‘The “White Woman’s Burden” in the “White Man’s Grave”: The Introduction of British Nurses in Colonial West Africa’, in Nupur Chaudhuri and Margaret Strobel (eds), Western Women and Imperialism: Complicity and Resistance (Bloomington: Indiana University Press, 1992), 177–88; Dale, *op. cit.* (note 4), 60–83.

70 M.A.M., *op. cit.* (note 65), 844.

71
*Ibid.*, 844.

72
*Ibid.*, 844.

73
*Ibid.*, 844.

74
*Ibid.*, 843.

75 A.V. Stewart, ‘Fighting the Pestilence: A Nurse’s Experiences in India’, *The Leisure Hour*, (August 1899), 637–43.

76
*Ibid.*, 637.

77
*Ibid.*, 638.

78 Healey, *op. cit.* (note 28), 1. Besides the cited evidence from the nurse’s semi-autobiographical periodical account, I noticed the term ‘sister’ being used to refer to her colleagues, both individually and as a collective noun (for instance, she refers to their accommodation as sisters’ quarters, or for self-reference as ‘we sisters’), in the personal letters of Nurse Dowson, which shall be discussed in the next section of this paper.

79 Stewart, *op. cit.* (note 75), 637.

80 Nineteenth-century indigenous medical systems in colonial Bombay were interwoven with local religious beliefs and practices. Attributing diseases to Gods, they were perceived as both their causes and cures. However, during the plague years, the colonial government dismissed these religio-medical approaches to diseases as superstitions, adding that they deterred the populace from accepting effective Western medical practices like vaccination. See David Arnold , Colonizing the Body: State Medicine and Epidemic Disease in Nineteenth-century India (Berkeley: University of California Press, 1993), 213 .

81 Pratt, *op. cit.* (note 7), 72, 74, 77, 84. Pratt argues that the European travellers’ colonial accounts suffered a rhetorical shift from traditions of survival literature to a sentimental mode in the late eighteenth century. Although the traveller occupies the central figure of the account, he is constructed as a non-interventionist European presence, and his expressions are anchored in his sensory experience.

82
*Ibid.*, 64.

83

Indira Ghose , Women Travellers in Colonial India: The Power of the Female Gaze (New Delhi: OUP, 1998), 5–9 .

84 Stewart. *op. cit.* (note 75), 637.

85
*Ibid.*, 638.

86 Pratt, *op. cit.* (note 7), 77.

87

Barbara N. Ramusack , ‘Cultural missionaries, maternal imperialists, feminist allies: British women activists in India, 1865-1945’ in Nupur Chaudhuri and Margaret Strobel (eds), Western Women and Imperialism: Complicity and Resistance (Bloomington: Indiana University Press, 1992), 119–36.

88
*Ibid.*, 133.

89 Stewart, *op. cit.* (note 75), 642.

90
*Ibid.*, 642–43.

91
*Ibid.*, 643.

92
*Ibid.*, 642.

93
*Ibid.*, 642.

94 Arnold, *op. cit.* (note 58), 125.

95
*Ibid.*, 125. Arnold notes how neither the Album nor the Newspapers showed the actual cremation rites being performed. He also adds that these rites, which were traditionally conducted on the banks of a river, could not be seen portrayed alongside any river in the plague archives as they were replaced by mass cremations during the plague. However, the images of cremation rites in this account, carried out on the banks of River Tapti, contest Arnold’s arguments.

96 Stewart, *op. cit.* (note 75), 643.

97
*Ibid.*, 642.

98
*Ibid.*, 643.

99

Susan L. Blake , ‘A woman’s trek: What difference does gender make?’, in Nupur Chaudhuri and Margaret Strobel (eds), Western Women and Imperialism: Complicity and Resistance (Bloomington: Indiana University Press, 1992), 19–34 .

100
*Ibid.*, 32.

101 Hester May Dowson, *Bombay During the Plague, 1897-98: Extracts from the letters of H.M.D.* (1898). (Provided by the British Library: India Office Records and Private Papers; accessed in July 2022.)

102

Clare Monagle , Carolyn James , David Garrioch , and Barbara Caine , European Women’s Letter-writing from the Eleventh to the Twentieth Centuries (Amsterdam: Amsterdam University Press, 2023), 210 .

103
*Ibid.*, 196.

104
*Ibid.*, 197.

105
*Ibid.*, 22, 198.

106 Although many women in the eighteenth and nineteenth centuries wrote letters intending them for public consumption through publication, women’s letter-writing was primarily understood as a practice of the private sphere. See Catherine Delafield , Women’s Letters as Life Writing 1840-1885 (New York: Routledge, 2020), 100 .

107

Klaus Stierstorfer , ‘General Introduction’, in Silke Strickrodt (ed.), Women Writing Home: 1700-1920: Female Correspondence Across the British Empire *, Vol* 1 (London: Routledge, 2016), 2 . Stierstorfer argues that the women’s letters written home from the colonies permeated British cultural history from the eighteenth to the twentieth centuries. They are valuable sources, owing to their predominantly private nature, biographic vividness, accessibility of style and often homely confidentiality within which they were produced.

108 Many of the letters written in an imperial context were written for a family audience, to be read aloud and shared. See Monagle et.al., *op. cit.* (note 102), 219.

109 Dowson, *op. cit.* (note 101), 11.

110
*Ibid.*, 6–7.

111
*Ibid.*, 18.

112
*Ibid.*, 14–15.

113
*Ibid.*, 5–6.

114

Ambalika Guha , ‘The “Masculine” Female: The Rise of Women Doctors in Colonial India, c.1870–1940.’ Social Scientist 44, 5/6 (2016), 49–64 .

115 Arnold, *op. cit.* (note 25), 21–22.

116 George and Lourdusamy, *op. cit.* (note 35), 356–7.

117

Sara Suleri , The Rhetoric of English India (Chicago: University of Chicago Press, 1996), 85 .

118 Ramusack, *op. cit.* (note 87), 132–4.

119

Nancy L. Paxton , ‘Complicity and resistance in the writings of Flora Annie Steel and Annie Besant’, in Nupur Chaudhuri and Margaret Strobel (eds.), Western Women and Imperialism: Complicity and Resistance (Bloomington: Indiana University Press, 1992), 159–60.

120 Dowson, *op. cit.* (note 101), 13.

121 Healey, *op. cit.* (note 28), 56; *Ibid.*, 21.

122
*Ibid.*, 17.

123
*Ibid.*, 18.

124
*Ibid.*, 19.

125
*Ibid.*, 34.

